# HIV Prevalence and Incidence in a Cohort of Women at Higher Risk for HIV Acquisition in Chókwè, Southern Mozambique

**DOI:** 10.1371/journal.pone.0097547

**Published:** 2014-05-19

**Authors:** Paul J. Feldblum, Sónia Enosse, Karine Dubé, Paulo Arnaldo, Chadreque Muluana, Reginaldo Banze, Aristides Nhanala, Joana Cunaca, Pai-Lien Chen, Merlin L. Robb, Ricardo Thompson

**Affiliations:** 1 FHI 360, Durham, North Carolina, United States of America; 2 Chókwè Health Research and Training Center/Centro de Investigação e Treino em Saúde de Chókwè (CITSC), National Institute of Health, Mozambique; 3 Institute for Global Health and Infectious Diseases (IGHID), University of North Carolina at Chapel Hill, Chapel Hill, North Carolina, United States of America; 4 United States Military HIV Research Program (MHRP), Henry M. Jackson Foundation for the Advancement of Military Medicine, Inc. (HJF), Bethesda, Maryland, United States of America; UCL Institute of Child Health, University College London, United Kingdom

## Abstract

**Background:**

Reliable HIV incidence estimates for Mozambique are limited. We conducted a prospective HIV incidence study as part of a clinical research site development initiative in Chókwè district, Gaza Province, southern Mozambique.

**Methods:**

Between June 2010 and October 2012, we recruited women at sites where women at higher risk of HIV infection would likely be found. We enrolled and tested 1,429 sexually active women in the screening phase and 479 uninfected women in the prospective phase. Participants were scheduled for 12+ months follow-up, when they underwent face-to-face interviews, HIV counseling and testing, and pregnancy testing. We observed a total of 373.1 woman-years (WY) of follow-up, with mean (median) of 9.4 (9.7) women-months per participant.

**Results:**

The prevalence of HIV was 29.4% (95% confidence interval [CI]: 27.0–31.8%). In multivariable logistic regression analysis, factors that remained significantly associated with prevalent HIV were: older age (OR: 0.6; 95% CI: 0.4–0.7), lower educational level (OR: 0.4; 95% CI: 0.3–0.7), and using hormonal contraception (OR: 0.6; 95% CI: 0.4–0.7) or condoms (OR: 0.5; 95% CI: 0.3–0.7). We observed an HIV incidence rate of 4.6 per 100 WY (95% CI: 2.7, 7.3). The HIV incidence was 4.8 per 100 WY (95% CI: 2.5, 8.3) in women aged 18–24 years, 4.5 per 100 WY (95% CI: 1.2, 11.4) in women aged 25–29 years and 3.2 per 100 WY (95% CI: 0.1, 18.0) in the 30–35 years stratum. None of the demographic factors or time-varying behavioral factors examined was significantly associated with incident HIV infection in bivariable analysis at p≤0.10.

**Conclusions:**

We found a high HIV incidence among sexually active young women in Chókwè, Mozambique. HIV prevention programs should be strengthened in the area, with more comprehensive reproductive health services, regular HIV testing, condom promotion, and messaging about multiple sexual partners.

## Introduction

Mozambique, located in southeast Africa, has one of the world’s highest HIV/AIDS burdens, with 1.2 million people infected at the end of 2011 [1]. HIV prevalence increased dramatically nationwide through the 1990s and has stabilized at a high rate. By 2009, 23 years after the first case of HIV was diagnosed there, the national adult HIV/AIDS prevalence was 11.5% [2], with the majority of new HIV infections acquired through heterosexual contact. According to 2009 national surveillance data, Gaza Province in the south had the highest HIV prevalence in the country at 29.9% among women 15–49 years old and 16.8% among men the same age (25.1% overall [2]. The percentage of women aged 15–49 years old that received an HIV test in the past year was 26%, compared to 10% of men of the same age [2].

HIV incidence data are scant in Mozambique. We conducted a cross-sectional screening and prospective cohort study of HIV in Chókwè, Gaza Province, southern Mozambique as part of a site development initiative to assess the feasibility of and build the capacity for HIV prevention research. We report here the HIV prevalence and incidence results and risk factors for HIV in a cohort of women recruited at venues where those at higher risk of HIV would likely be found.

## Methods

The study was conducted at the Chókwè Health Research and Training Center (CITSC) in Chókwè city, a research unit affiliated with the Mozambican National Institute of Health (INS). The study was done in collaboration with FHI 360 and the United States Military HIV Research Program (MHRP). Chókwè district is located in Gaza Province along the Limpopo River in the southern region of the country, about 220 kilometers northwest of Maputo, and is home to about 200,000 people, nearly half of whom reside in Chókwè city, where recruitment and enrollment were focused. The city is the center for the surrounding agricultural area which includes subsistence farmers as well as a major irrigation system supporting large rice production enterprises.

### Study Design and Population

Women identified as potentially at higher risk of HIV infection were invited to enroll in the study. Over 1,400 women were screened in a cross-sectional study that served as the screening phase for the prospective study. We invited HIV-negative women to enroll in the prospective phase, which involved monthly follow-up visits for up to 12 months.

The primary objective of the study was to measure prospectively the incidence of HIV infection. A secondary objective was to assess the site’s ability to enroll and retain the cohort for one (and later two) year(s). Inclusion criteria were: women aged 18 to 35 years; HIV-negative by rapid testing; sexually active in the last month; willing to adhere to study visit requirements; and planning to reside in Chókwè for the duration of the study. Women were excluded if they had a medical condition that precluded their participation in the study. Pregnancy was not an exclusion criterion.

Mid-way through the study, we amended the protocol to increase the study size, lengthen the total duration of follow-up to 24 months per participant, and change the frequency of follow-up visits to every three months. The study size was increased to provide more precision around possible lower incidence ranges, and the follow-up schedule was modified to mimic better the likely design of future HIV vaccine trials. But the study was terminated early for logistical and financial reasons unrelated to study performance.

### Recruitment Procedures

Prior to study initiation, community sensitization activities were carried out, consisting of informational meetings with the Chókwè district and health authorities, numerous *bairro* (neighborhood) leaders and community members. Potential participants were recruited in various community venues in Chókwè city (markets, nightclubs, bars, hotels), as well as secondary schools, where we would be likely to find young women who engage in risky sexual behavior. Interested women were approached by field workers who explained more about the study objectives and procedures. Volunteers were given an appointment card and invited to visit the Chókwè Health Research and Training Center (CITSC) for screening. Informed consent was administered, and HIV and pregnancy testing along with interviewing ensued. To enhance follow-up in the prospective phase, we contacted participants before their scheduled visit dates, as well as after missed visits.

### Data Collection and Study Procedures

Data collection took place between June 2010 and October 2012 at the CITSC adjacent to the Chókwè District Hospital. We collected baseline, demographic, behavioral, and clinical data and performed counseling and rapid testing for HIV and pregnancy. Counseling and HIV and pregnancy testing were repeated at each follow-up visit, along with behavioral interviews. Participants received condoms free of charge at each visit, and were given 150 Meticais reimbursement (about USD $5) to compensate for missed income and transport costs. Women found to be HIV-infected, pregnant or suspected of having a sexually transmitted infection (STI) were referred for care in the nearby public clinics.

Data were double-entered on-site into a ClinTrial database (Oracle Health Sciences, Redwood Shores, CA, USA) and transmitted to the FHI 360 server using Title 21 Code of Federal Regulations Part-11 compliant Citrix 12.3 software.

### Laboratory Testing

We performed serial rapid HIV testing first with the Determine HIV-1/2 test (Alere Medical Co. Ltd., Chiba, Japan), with reactive results followed with Uni-Gold Recombigen HIV test (HIV Trinity BioTech PLC, Bray, Ireland), per Mozambique’s national HIV testing algorithm. We used parallel SD Bioline HIV-1/2 version 3.0 test (Standard Diagnostics Inc., Kyonggi-do, Korea) and Vironostika HIV-1 ELISA testing (Uni-Form II Plus 0 test, BioMerieux BV, Boxtel, The Netherlands) to resolve discrepant results. We stored aliquots of plasma at −80°C to allow confirmation of possible HIV seroconversion events.

Incident HIV infection was defined as two positive rapid HIV antibody tests or one positive HIV rapid test confirmed by positive ELISA Vironostika test, after negative results at the previous visit. All incident HIV infections were confirmed by quantitative HIV-1 RNA PCR with stored specimens at the national reference laboratory of the INS in Maputo. Urine pregnancy testing was done using a rapid human chorionic gonadotropin (hCG) test (Healthease Preg n Care, NEOMED IPA, Tzaneen, South Africa).

### Statistical Analysis

The prospective cohort study was designed to enroll approximately 400 women and observe at least 380 person-years of follow-up to conclude that the HIV incidence is no less than 2.1% (two-sided α = 0.05) if the observed incidence was 4.2 per 100 person-years. The mid-study protocol amendment intended to demonstrate the feasibility of future HIV vaccine trials called for an increased study size of 735 participants, which was not achieved due to funding constraints.

We summarized baseline variables using descriptive statistics expressed as mean or median for continuous variables and percentages for categorical variables. In the prospective phase, HIV-negative participants were censored on the date of the last follow-up visit. For HIV seroconvertors, we estimated the date of seroconversion as the midpoint between the last HIV RNA PCR negative and the first HIV RNA PCR positive test result during the follow-up period.

After measuring HIV prevalence in the screening phase, we used bivariable logistic regression to assess determinants of prevalent HIV infection; all factors associated with HIV-1 infection at p≤0.10 in bivariable analyses were included in a multivariable logistic regression model. We estimated HIV incidence using the number of confirmed HIV seroconversions per 100 woman-years (WY) of follow-up, and calculated the 95% confidence interval (CI) of the incidence rate using exact methods under the assumption that the number of HIV infections follows a Poisson distribution. We used Cox regression analysis to calculate bivariable and multivariable hazard ratios (HRs) and their 95% CIs to evaluate the associations between incident HIV infection and baseline characteristics and time-varying risk behaviors. All data analyses were conducted using SAS version 9.3 (Cary, North Carolina).

### Ethical Considerations

The National Committee for Bioethics/Comité Nacional de Bioética para a Saúde (CNBS) in Maputo, Mozambique, the FHI 360 Protection of Human Subjects Committee (PHSC), and the Division of Human Subjects Protection (DHSP) of the Walter Reed Army Institute of Research (WRAIR) reviewed and approved the study protocol and other essential documents. Written informed consent was obtained from all participants prior to initiating study procedures.

## Results

### Study Population

The study screened 1,429 women in the cross-sectional screening phase. 479 of the 1,009 HIV-negative women were eligible and enrolled, and contributed HIV test data in the prospective phase (enrollment ended at this point). We observed a total of 373.1 WY of follow-up, with mean (median) of 9.4 (9.7) women-months per participant. The overall completion rate, including women who seroconverted, was 70.3%; 26.6% of the women were lost to follow-up and 3.2% discontinued early.

More than half (60.5%) of women enrolled in the screening phase were between 18–24 years old; the median age was 23 (Table 1). Educational attainment was modest, and over half (53.7%) of the women were unemployed. Around one third (39.2%) of the women were married. Nearly half (45.6%) reported 2 or more sexual partners in the last month, with a smaller number (14.2%) reported having had a new sexual partner in the last month. Half (51.8%) of the screening phase enrollees used no contraception at baseline; the most commonly used method was oral contraceptives (20.9%), and one in seven (13.9%) women reported condoms as their contraceptive method. For all of the above factors, the characteristics of the sub-set of women who entered the prospective phase and contributed follow-up data (N = 479) were similar (Table 1).

**Table 1:** Baseline features of women entering Cross-sectional and Prospective Phases, Chókwè, Mozambique.

	Cross-Sectional Phase N (%^1^)	Prospective Phase N (%^1^)
	1429	479
**Age**		
Median (IQR)	23 (20–27)	22 (19–26)
18–24	864 (60.5)	323 (67.4)
25–29	369 (25.8)	115 (24.0)
30+	196 (13.7)	41 (8.6)
**Education**		
None	366 (25.6)	82 (17.1)
Primary school (grade 1–5)	438 (30.7)	139 (29.0)
Secondary school (grade 6–9)	439 (30.7)	175 (36.5)
High school and higher	186 (13.0)	83 (17.3)
**Employment**		
Unemployed	767 (53.7)	248 (51.8)
Employed	147 (10.3)	52 (10.9)
Other	514 (36.0)	179 (37.4)
Missing	1 (0.1)	–
**Type of work**		
Office work, sales	285 (19.9)	86 (18.0)
Manual labor, food service	30 (2.1)	6 (1.3)
Student	150 (16.9)	125 (26.1)
Sex worker	2 (0.2)	0 (0.0)
Other	54 (3.8)	14 (2.9)
Missing	767 (53.7)	248 (51.8)
**Marital status**		
Unmarried	762 (53.3)	234 (48.9)
Married	560 (39.2)	206 (43.0)
Other	107 (7.5)	39 (8.1)
**Number of live births (mean (median))**	1.5 (1.0)	1.4 (1.0)
**Number of sexual partners in the last 1 month**		
Median (IQR)	1 (1–2)	1 (1–2)
<2	778 (54.4)	322 (67.2)
2+	651 (45.6)	157 (32.8)
**Number of NEW sexual partners in the last 1 month**		
0	1226 (85.8)	441 (92.1)
1	164 (11.5)	31 (6.5)
2+	39 (2.7)	7 (1.5)
**Current contraceptive**		
None	740 (51.8)	219 (45.7)
Oral	298 (20.9)	108 (22.5)
Injectable	182 (12.7)	62 (12.9)
Condoms	198 (13.9)	89 (18.6)
Other/Missing	11 (0.8)	1 (0.2)

1 Column percentage.

IQR = interquartile range.

### Prevalence of HIV in Cross-sectional Screening Phase

The prevalence of HIV was 29.4% (95% CI: 27.0–31.8%). Older age was strongly associated with prevalent HIV infection in bivariable analysis, as was lower educational attainment (Table 2). Several sexual risk behaviors were associated with prevalent HIV, including having multiple partners (OR: 2.0; 95% CI: 1.4–2.8; p<0.001), a new partner in the last month (OR: 1.7; 95% CI: 1.2–2.3; p = 0.001), and reporting vaginal sex without a condom in the last 7 days with a partner that was not considered primary (OR: 1.5; 95% 1.2–1.9; p<0.001). Using hormonal contraception or condoms for contraception was associated with lower HIV infection risk (OR: 0.6; 95% CI: 0.5–0.8; p<0.001 and OR 0.3; 95% CI: 0.2–0.5; p<0.001), respectively (Table 2). In multivariable analysis, factors that remained statistically significant after controlling for other variables included: older age (OR: 0.6; 95% CI: 0.4–0.7; p<0.001), lower educational level (OR: 0.4; 95% CI: 0.3–0.7; p<0.001), and using hormonal contraception (OR: 0.6; 95% CI: 0.4–0.7; p<0.001) or condoms (OR: 0.5; 95% CI: 0.3–0.7; p<0.001).

**Table 2:** Factors associated with prevalent HIV infection for women enrolled in the Cross-sectional Phase, Chókwè, Mozambique (N = 1429).

	Bivariable Analysis		Multivariable Analysis	
	Odds Ratio (95% CI)	p-value	Odds Ratio (95% CI)	p-value
**Age**				
< = 24	0.5 (0.4, 0.6)	<0.001	0.6 (0.4, 0.7)	<0.001
>24	Reference		Reference	
**Education**				
> = Grade 10	0.3 (0.2, 0.5)	<0.001	0.4 (0.3, 0.7)	<0.001
Grade<10	Reference		Reference	
**Sex worker/transactional sex**				
Yes	1.5 (0.9, 2.4)	0.15		
No	Reference			
**>2 sex partners in last month**				
Yes	2.0 (1.4, 2.8)	<0.001	1.5 (1.0, 2.3)	0.07
No	Reference		Reference	
**New sex partner(s) last month**				
Yes	1.7 (1.2, 2.3)	0.001	1.2 (0.8, 1.7)	0.45
No	Reference		Reference	
**Vaginal sex without using condom in last 7 days with primary partner**				
Without using	0.9 (0.7, 1.1)	0.22		
Using	Reference			
**Vaginal sex without using condom in last 7 days with other partner**				
Without using	1.5 (1.2, 1.9)	<0.001	1.1 (0.9, 1.5)	0.43
Using	Reference		Reference	
**Baseline contraception**				
Hormonal method	0.6 (0.5, 0.8)	<0.001	0.6 (0.4, 0.7)	<0.001
Condom	0.3 (0.2, 0.5)	<0.001	0.5 (0.3, 0.7)	<0.001
Other	2.7 (0.8, 9.6)	0.13	3.2 (0.9, 12.2)	0.09
Nothing	Reference		Reference	

### HIV Incidence in Prospective Phase

We observed 17 seroconversions in the prospective cohort, an overall HIV incidence rate of 4.6 per 100 WY of follow-up (95% CI: 2.7, 7.3). The HIV incidence decreased slightly with age: 4.8 per 100 WY of follow-up (95% CI: 2.5, 8.3) in women aged 18–24 years; 4.5 per 100 WY of follow-up (95% CI: 1.2, 11.4) in women aged 25–29 years; and 3.2 per 100 WY of follow-up (95% CI: 0.1, 18.0) in the 30–35 years stratum.

When we examined factors associated with incident HIV infection in Cox models, none of the variables was significantly associated in bivariable analysis at the p≤0.10 level (Table 3). We therefore did not conduct a multivariable analysis. The direction of the weak bivariable associations generally conformed to those seen in the logistic regression findings for the screening phase.

**Table 3:** Factors associated with incident HIV for women enrolled in the Prospective Phase, Chokwe, Mozambique (N = 479).

	Bi-variable Analysis	
	Unadjusted HR (95% CI)	p-value
**Age**	1.0 (0.9,1.1)	0.67
**Education**		
≥ Grade 10	0.6 (0.1,2.8)	0.56
<Grade 10	Reference	
**Sex worker/transactional sex**		
Yes	3.4 (0.5,25.9)	0.23
No	Reference	
**Marital status**		
Yes	0.4 (0.1,1.2)	0.10
No	Reference	
**New partner(s) in last month**		
Yes	0.7 (0.1,4.9)	0.68
No	Reference	
**Vaginal sex without using condom in last 7 days with primary partner**		
without using	0.7 (0.3,1.8)	0.45
Using	Reference	
**Vaginal sex without using condom in last 7 days with other partner**		
without using	2.5 (0.8,7.8)	0.10
Using	Reference	
**Baseline contraception**		
Hormonal method	0.4 (0.1,1.3)	0.14
Condom	0.4 (0.1,2.0)	0.28
Other	0.0 (0.0,0.0)	0.99
Nothing	Reference	

HR = hazard ratio.

## Discussion

These data represent some of the first longitudinal HIV incidence results from Mozambique. We detected a high HIV prevalence (29.4%) and incidence (4.6 per 100 WY), despite consistent risk reduction counseling and condom availability at all study visits. Our findings confirm the magnitude of the HIV epidemic in the southern region of Mozambique: the HIV prevalence in the study is almost exactly the same as that found in the last national surveillance survey among women aged 15–49 years old (29.9%) [2].

The HIV prevalence and incidence we measured in the southern city of Chókwè were similar to those in a related study conducted in the larger central Mozambican city of Beira [3]–[4], although the Beira incidence rate was even higher at 6.5 per 100 WY [4]. A large prospective study of postpartum women in southern Mozambique also found a substantial HIV incidence rate of 3.2 per 100 WY overall, highest in the youngest women of 18–19 years [5]. The incidence rate among participants in that study from Gaza Province, where Chókwè is located, was slightly higher at 3.6 per 100 WY.

Our study had several limitations. Foremost was the relatively high loss to follow-up of 27%, despite vigorous tracing efforts. Chókwè represented a particularly challenging environment for retention activities, due to long distances to cover with the low population density, and an episode of severe flooding during the study. Besides travel obstacles, frequent reasons for missing follow-up visits were concerns about the blood draws and partner objection to participant return for HIV testing. Part-way through the study, we revised the design to follow participants for as long as 24 months, and some women were indeed followed for more than 12 months (none for 24). But the study was terminated for financial reasons before most women could surpass 12 months, and the mean and median woman-months of observation were 9 and 10 months respectively. Also, all participants who were ultimately lost-to-follow-up made at least one visit and contributed person-time and HIV data to the analysis.

Early study termination meant that we detected fewer new HIV infections and had lower power to detect associations in the Cox regression analyses. With 17 seroconversion events, we had power to detect only strong associations, which likely explains our inability to detect any factors significantly associated with new HIV infection.

It may be that the Chókwè HIV epidemic is a highly complex one associated with migrant labor patterns, placing all sexually active women at higher risk for HIV acquisition. Although close to one third of the study participants were married (36.8%), the HIV epidemic in Chókwè cannot be discussed without taking the male migrant labor pattern into account as this disrupts family structures in Chókwè. The majority of Mozambican migrant mineworkers, most of whom work in neighboring South Africa, come from southern Mozambique, with approximately 40% of them from Gaza province, mainly Xai-Xai (the provincial capital) and Chókwè [6]. Other relevant factors that were outside the scope of our study include a poor health care infrastructure, widespread poverty, a lengthy civil war (1977 to 1992) and proximity to countries with substantial HIV epidemics [7].

Our study found a high prevalence of multiple sexual partnerships at screening (45.6%) and at prospective enrollment (32.8%), defined as reporting more than one partner in the past month. These data appear inconsistent with the national HIV surveillance data, where 1% of women age 15–49 reported having had two or more sexual partners in the past 12 months [2]. But by design, our study aimed at recruiting women at higher risk for HIV acquisition and focused recruitment on venues known for sex exchanges (i.e. bars, markets, etc.). We found that baseline HIV prevalence was associated with multiple partners (OR: 2.0) and having had a new sexual partner in the last month (OR: 1.7). Given the complexity of the HIV epidemic in Chókwè, it seems likely that multiple partnerships help fuel HIV transmission, especially against a backdrop of high HIV prevalence.

We also found low rates of condom use for family planning at screening (13.9%) and at prospective enrollment (18.6%). These data are similar to rates reported for the general population of women aged 15–49 years old (9.5%) [2]. Fertility desires may contribute to low condom use in this population [8]. Young women wishing to become pregnant should be counseled on approaches to reduce their risk of HIV infection. The data further underscore the need for intensive HIV prevention programming with individuals having multiple partners and low condom use in Chókwè.

The study demonstrates a considerable HIV incidence in this Mozambican city. The force of HIV infection remains strong in those places in Mozambique where incidence has been measured [4]. Our results point to the need for improved reproductive health services, and more vigorous educational efforts and prevention programming in Chókwè district.
